# Complete pathological response to pembrolizumab in pretreated pancreatic acinar cell carcinoma

**DOI:** 10.1007/s00432-024-05841-z

**Published:** 2024-07-11

**Authors:** Valeria Merz, Francesca Maines, Stefano Marcucci, Chiara Sartori, Michela Frisinghelli, Chiara Trentin, Dzenete Kadrija, Francesco Giuseppe Carbone, Andrea Michielan, Armando Gabbrielli, Davide Melisi, Mattia Barbareschi, Alberto Brolese, Orazio Caffo

**Affiliations:** 1 Department of Medical Oncology, Santa Chiara Hospital, APSS, L.Go Medaglie d’Oro,9, 38122 Trento, Italy; 2 Digestive Molecular Clinical Oncology Research Unit, Università degli Studi di Verona, Verona, Italy; 3 Department of General Surgery and HPB Unit, Santa Chiara Hospital, APSS, Trento, Italy; 4 Department of Laboratory Medicine - Pathology Unit, Santa Chiara Hospital, APSS, Trento, Italy; 5 Gastroenterology and Digestive Endoscopy Unit, Santa Chiara Hospital, APSS, Trento, Italy; 6 Center for Medical Sciences (CISMed), University of Trento, Trento, Italy

**Keywords:** Pancreatic cancer, Immunotherapy, Acinar cell carcinoma, pCR, Surgical resection, Neoadjuvant therapy

## Abstract

**Background:**

Therapeutic approach used for pancreatic ductal adenocarcinoma is usually translated also for the rarer acinar counterpart, which shows a different mutational landscape nevertheless. While dMMR/MSI-H status is rare in the ductal histotype, it appears to be more prevalent in pancreatic acinar cell carcinoma (PACC).

**Case presentation:**

We report the case of a patient with locally advanced MSI-H PACC in whom the treatment with the anti-PD-1 pembrolizumab, administered as third line, made possible surgical resection, achieving even an exceptional pathological complete response.

**Conclusions:**

Treatment of PACC should be tailored based on the peculiar molecular features that distinguish PACC from ductal adenocarcinoma. Evaluation of potentially therapeutically targetable alterations should be mandatory in case of PACC diagnosis.

## Introduction

Pancreatic acinar cell carcinoma (PACC) constitutes only 1–2% of exocrine pancreatic tumors, despite acinar cells being predominant in pancreatic tissue compared to the ductal counterpart (Klimstra 2007).

The incidence of PACC has a bimodal distribution with peaks at 8–15 and 60 years (Lack et al. 1983). Males are affected three times more often (Schmidt et al. 2008).

Patients diagnosed with PACC typically manifest non-specific symptoms, such as weight loss, pain, nausea, and vomiting, while jaundice is relatively uncommon (Calimano-Ramirez et al. 2022). Lipase hypersecretion can cause the "Schmid's Triad", characterized by subcutaneous fat necrosis, polyarthralgia, and eosinophilia (Schmid 1957).

PACC tumors are solid, well-circumscribed, and often bulky tumors, and are most frequently localized in the pancreatic head (Qu et al. 2022). Larger lesions may present necrosis, cystic changes and intratumoral haemorrhage. Approximately half of tumors show local invasion of the surrounding organs and a similar proportion of patients present with metastatic dissemination, more often to the liver, peritoneum and distant lymph nodes (Rosa et al. 2012; Takahashi et al. 2021). Microscopically, PACC is characterized by minimal stroma and a predominant acinar or solid architecture. Immunohistochemical staining for pancreatic enzymes, including trypsin, chymotrypsin, elastase, or lipase, supports the histological diagnosis (Klimstra et al. 1992). Bcl-10 can also be used for identifying acinar cell differentiation.

Patients affected by PACC may exhibit elevated levels of carbohydrate antigen 19–9 (CA-19.9), alpha-fetoprotein (AFP) and carcinoembryonic antigen (CEA), besides serum lipase increase (Calimano-Ramirez et al. 2022).

Different molecular pathways are involved in PACC tumorigenesis compared to pancreatic ductal adenocarcinoma (PDAC). Key PDAC carcinogenesis genes are less frequently mutated in PACC, such as p53 (13.7%), SMAD4 (15.1%), p16 (11.6%), or rarely altered, such as KRAS (3.8%) (Florou et al. 2023; Moore et al. 2001; Wilde et al. 2011; Jiao et al. 2014). Activation of the Wnt pathway, including APC inactivating mutations and CTNNB1 activating mutations, has been reported in about 20% of cases (Abraham et al. 2002). Chromosomal instability also characterizes PACC, partly explaining reported resistance to therapy (Jiao et al. 2014; Butturini et al. 2011). Notably, more than one-third of PACC exhibit potentially therapeutically targetable alterations, including BRCA1, BRCA2, PALB2, ATM, BAP1, BRAF and ALK (Jiao et al. 2014; Kryklyva et al. 2019). Some series report over half of PACC cases harbouring germline pathogenic variant affecting homologous recombination and DNA damage response genes (Mandelker et al. 2023). BRAF and RAF1 alterations, observed in about 23% of PACC, are mutually exclusive with inactivating mutations in DNA repair genes (Chmielecki et al. 2014). A variable proportion ranging from 2.1% to 14% of PACC exhibits microsatellite instability (MSI)/defective mismatch repair (dMMR) (Ikezawa et al. 2023; Luchini and Scarpa 2023).

The prognosis of this rare pancreatic cancer subtype is generally better than that of PDAC, with a 5-year survival rate of about 21–43% (Ikezawa et al. 2023). Surgical resection is recommended for localized disease and is associated with longer survival in various series (Holen et al. 2002; Distler et al. 2009).

Limited data guide optimal PACC treatment. In the absence of randomized trials for this subtype, patients with advanced PACC are typically treated with the same chemotherapeutic regimens as PDAC. Some retrospective evidence suggests higher response rates to fluoropyrimidines, particularly in oxaliplatin-based regimens, compared to gemcitabine-based therapies (Butturini et al. 2011; Lowery et al. 2019; Kruger et al. 2016; Yoo et al. 2017). The role of adjuvant treatment remains controversial, and no guidelines are available. Neoadjuvant strategies also lack prospective studies.

Genomic profiling evidence supports a high fraction of druggable targets in PACC. Tailored approaches, such as BRAF, ALK and NTRK inhibition, have shown generally good results (Florou et al. 2023; Li et al. 2018; Cramer et al. 2020; Gaule et al. 2022; Gupta et al. 2021).

Here, we present the first reported case of a patient with microsatellite instable PACC achieving a pathological complete response to anti-PD1 therapy.

## Case Presentation

A 69-year-old man presented with abdominal pain, dyspepsia and hyporexia, leading to diagnosis of PACC. His medical history included hypertension and hypercholesterolemia, and he was an active smoker (50 pack-years). Family history indicated unspecified tumors in his grandmother and uncle.

**Fig. 1:**
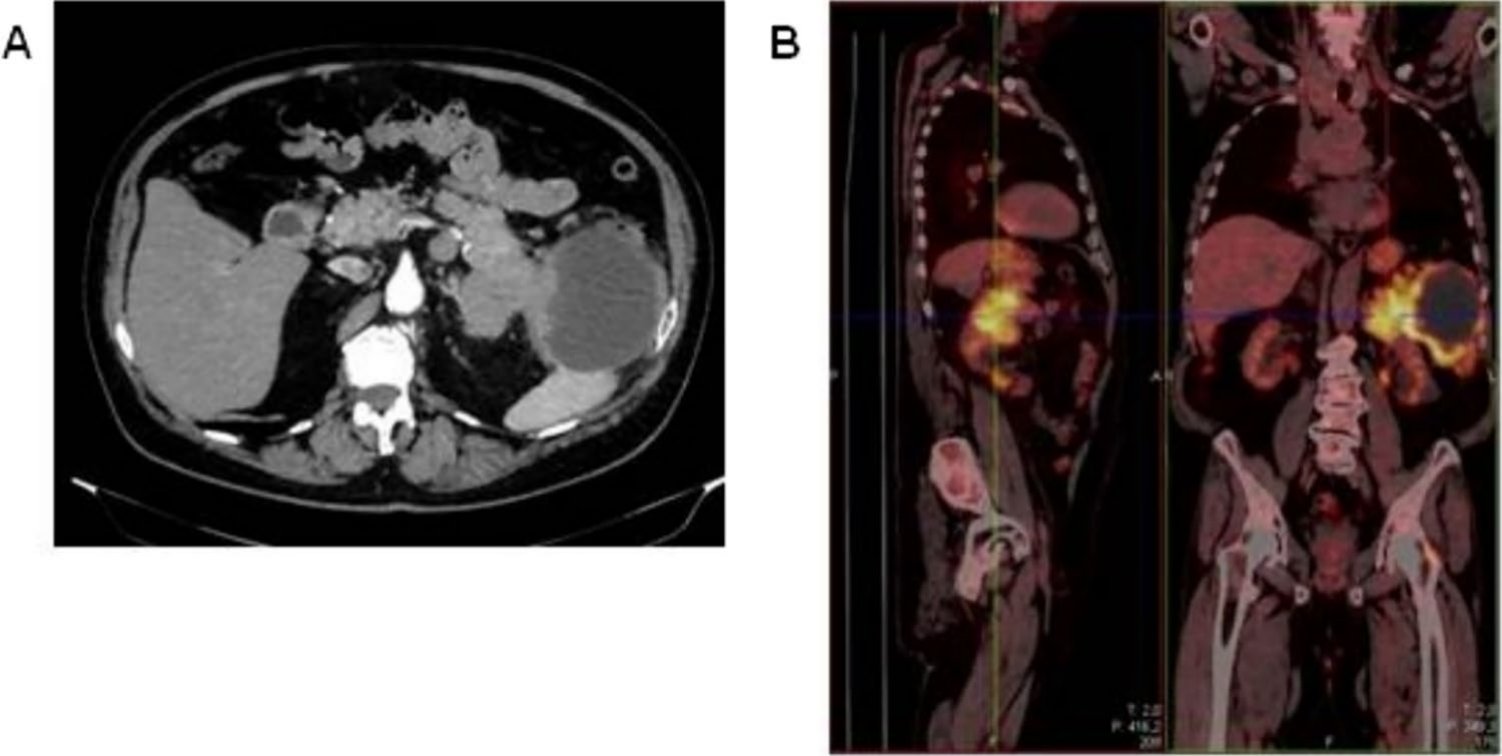
CT (**A**) and PET (**B**) scans at baseline

**Fig. 2:**
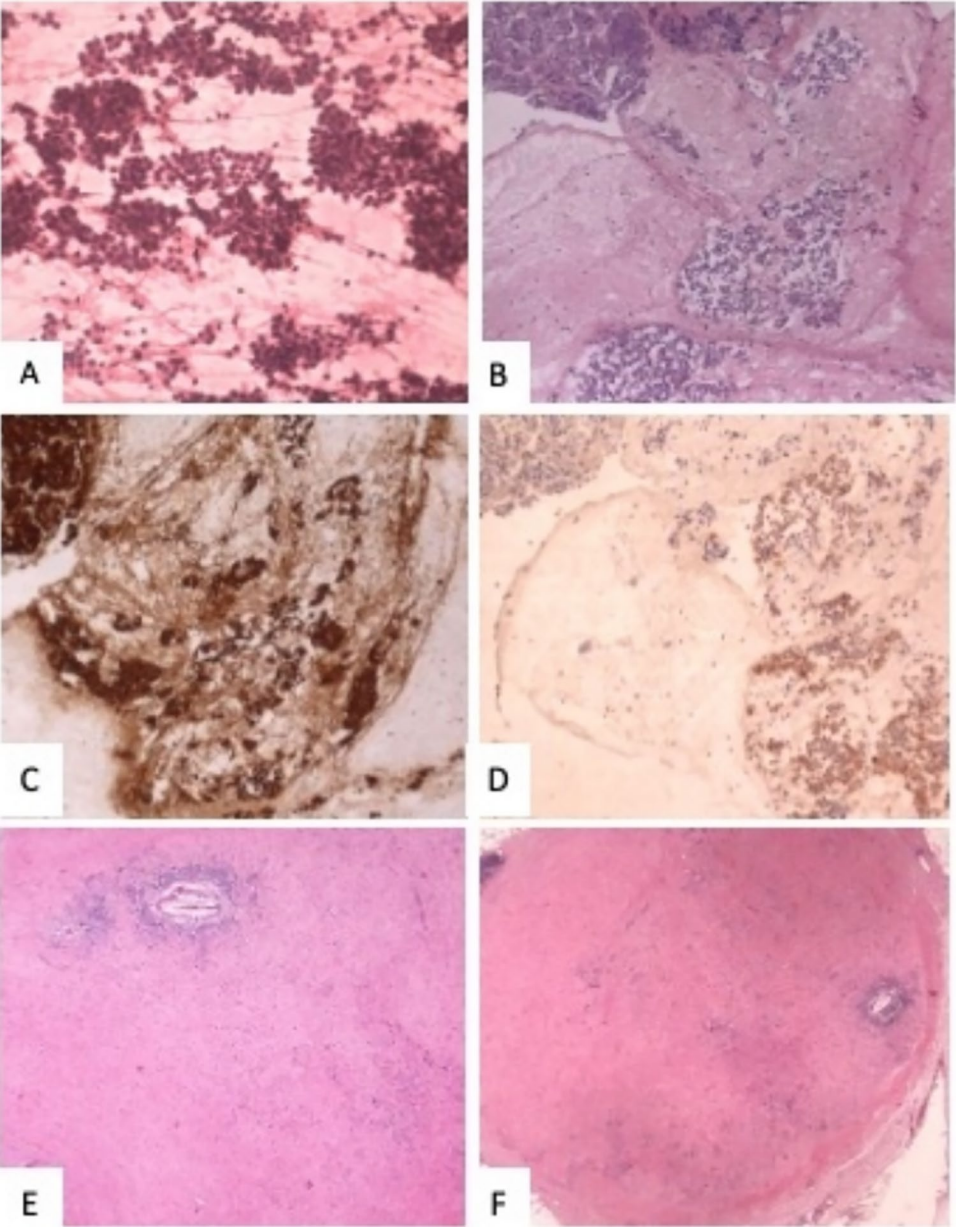
Pathological features of the tumor. **A–D** cytologic specimen at the time of diagnosis; **A** Hypercellular cytologic smear (Hematoxylin–eosin rapid stain, original magnification 40X). **B, D** Cell block showing numerous neoplastic cell with round nuclei and eosinophilic cytoplasm; on the left normal pancreatic parenchyma (**B** Hematoxylin–eosin, original magnification 10X; **C** Bcl-10, original magnification 10X; **D** Synaptophysin, original magnification 10X). **E–F**: surgical specimen. **E** Tumor bed (Hematoxylin–eosin, original magnification 4X). **F** Lymph node with response to therapy (Hematoxylin–eosin, original magnification 2X). (magnification, × 20)

Computed tomography (CT) revealed confluent masses with a central hypodense component, occupying an area of 15 cm in the pancreatic tail. This lesion appeared not dissociable from the ascending colon, bordered the large gastric curvature, the left adrenal gland, the spleen and the abdominal wall (Fig. 1A). Gross locoregional lymphadenopathies were shown. Fluorodeoxyglucose (FDG)-positron emission tomography (PET) confirmed an extensive hypermetabolic infiltrating neoplastic mass, with locoregional lymph nodal metastases (Fig. 1B). The patient underwent endoscopic ultrasonography with fine-needle aspiration (EUS-FNA). The cytological examination revealed the diagnosis of pancreatic acinar adenocarcinoma, supported by immunohistochemical positivity for BCL-10 and negativity for chromogranin A and synaptophysin (Fig. 2A–D). CEA resulted over the upper limit, while CA-19.9 and AFP showed normal levels.

After multidisciplinary evaluation, the patient was offered a neoadjuvant treatment with combined chemotherapy with oxaliplatin, irinotecan, folinic acid and fluorouracil (mFOLFIRINOX). After six chemotherapy courses, an imaging re-evaluation with CT scan and PET revealed a stable disease. Given the neoadjuvant intent of the treatment and considering the increasing CEA levels, the patient was switched to a combination regimen with gemcitabine plus nab-paclitaxel. Unfortunately, after three months a progression of primary tumor was detected by the imaging. The patient worsened, showing asthenia, dysphagia and hyporexia.

Given the absence of other efficacious therapies and the proportion of targetable alterations in PACC, the patient was candidate to next generation sequencing (NGS) analysis through liquid biopsy. FoundationOne Liquid CDx assay revealed MLH1 mutation and MSI-high status. Thirty-two mutations per Mb was the blood tumor mutational burden. A complete report of the NGS assay is reported in Table 1.

**Table 1:** Gene alterations identified with NGS assay

Altered gene	Gene alteration	VAF%
PI3KCA	E545D	4.2
ARID1A	Q1519fs*8	0.88
CTNNB1	D32Y	8.5
PTCH1	R1308fs*64	13.0
RAF1	rearrangement intron 7	4.0
ASXL1	G645fs*58	14.2
BCORL1	P1681fs*20	1.1
BRD4	Q256fs*27	8.9
CDH1	splice site 163 + 1G > A	0.61
CREBBP	splice site 3836 + 1G > A	7.5
JAK1	K860fs*16	0.46
MLH1	E178fs*24	8.8
RB1	R73fs*36	10.1
TP53	R181H A138V E294fs*51	11.3 7.6 0.85

*VAF%* variant allele frequency percentage

**Fig. 3:**
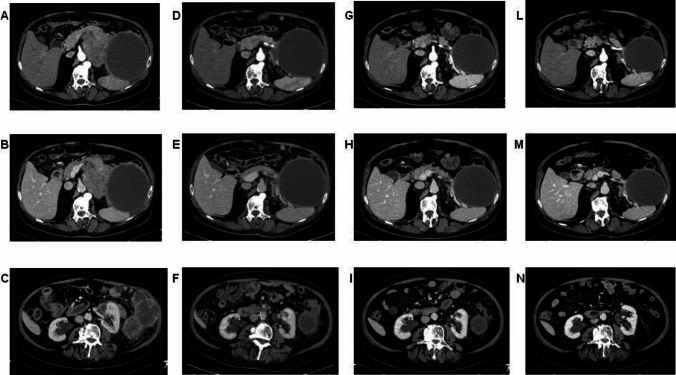
CT scans after two lines of chemotherapy, before the start of pembrolizumab (**A, B, C**). CT scans after 3 months (**D, E, F**), after 6 months (**G, H, I**) and after 10 months (**L, M, N**) of pembrolizumab

Given its activity in patients with MSI, pembrolizumab can be considered in these cases. The agnostic use of this immune checkpoint inhibitor is recognized by FDA, but not by EMA. Although in Italy pembrolizumab use in MSI patients with pancreatic cancer is possible only in off-label indication, it was proposed to this patient. After the first administration symptoms reported by the patient improved already and CEA levels rapidly dropped. The CT scan after 4 administrations of pembrolizumab demonstrated size reduction of the polylobate mass and of the solid-cystic component (Fig. 3). The subsequent CT scans after 10 and 15 cycles revealed further response (Fig. 3). Given the clinical improvement and both radiological and biochemical response, after multidisciplinary evaluation, the patient was proposed for surgery after 17 cycles of pembrolizumab. He underwent left subtotal splenopancreatectomy with extended resection of the retroperitoneum and of the left adrenal gland (radical antegrade modular pancreatosplenectomy, RAMPS). The histological examination of the surgical specimen demonstrated a pathological complete response (pCR), with 0 tumor regression grade according to modified Ryan score (Fig. 2E). Twenty lymph nodes were removed and six of them showed response to therapy (Fig. 2F). A large area of dense and loose fibrosis was found up to the capsule of the pseudocyst.

At the time of the present report, six months after surgery, the patient is alive and disease-free, undergoing periodic imaging evaluations.

The patient performed a genetic testing because of the somatic MLH1 mutation observed. However, Lynch syndrome was not detected, and no germinal alterations have been identified in a panel of 25 genes.

## Discussion

Currently, main international guidelines do not recommend performing NGS in pancreatic cancer, including in subtypes such as PACC (Mosele et al. 2020). However, PACC differs from the more common PDAC in its genomic profile, with a significant proportion of PACC exhibiting potentially targetable genetic alterations or MMR alterations (Florou et al. 2023; Chmielecki et al. 2014).

Based on this evidence, we performed NGS in our patient with advanced pre-treated PACC. Despite the failure of two chemotherapeutic regimens, the disease remained localized, prompting the search for an active therapy able to cytoreduce the mass. The liquid biopsy detected an MSI status determined by MLH1 mutation. Consequently, we opted for pembrolizumab, resulting in a rapid radiological response with improvement in clinical conditions. The most compelling result was the absence of tumor cells in the histologic examination of the surgical specimen.

MSI status can be tested by immunohistochemistry for mismatch repair proteins MLH1, PMS2, MSH2, and MSH6, PCR-based micro-satellite testing or NGS-based approaches (Luchini and Scarpa 2023). While dMMR/MSI-H status is rare in PDAC (1–2%), it appears more prevalent in PACC, ranging from 2.1 to 14% (Luchini and Scarpa 2023; Rosa et al. 2015). Available data are resumed in Table 2.

**Table 2:** Studies reporting dMMR/MSI prevalence in PACC

Ref	Number of patients analyzed for MSI	Proportion of MSI-H
Abraham et al. (2002)	13	7%
Liu et al. (2014)	36	14%
Bergmann et al. (2014)	42	4.8
Florou et al. (2023)	51	2.1
Sakakida et al. (2023)	44	2.6

Tumors with MSI/dMMR tumors are known to be sensitive to immunotherapeutic agents. In the 2023 U.S. Food and Drug Administration (FDA) granted full approval of pembrolizumab for the treatment of unresectable or metastatic MSI-H or dMMR solid tumors that have progressed following prior treatment (FDA 2023). Although the European Medicines Agency (EMA) did not provide a similar agnostic indication, this immune checkpoint inhibitor is approved for some subgroups of MSI or dMMR tumors (https:, , www.ema.europa.eu, en, documents, product-information, keytruda-epar-product-information_en.pdf. xxxx).

The FDA’s approval of pembrolizumab was based on data from patients with MSI-H or dMMR cancer enrolled in five multicenter, single-group clinical trials (Lemery et al. 2017). Among 59 patients with a cancer different that colorectal cancer, six had a pancreatic cancer with a response rate 83%.

Despite the low mutational load and the immune milieu limiting the activity of immunotherapy in pancreatic cancer, MSI pancreatic tumors exhibit a higher infiltration of CD8 + T cells and higher PD-1 and PD-L1 expression compared to the microsatellite stable ones (Ghidini et al. 2021). The majority of MSI/dMMR PDAC also show high TMB (Hu et al. 2018). Nevertheless, high TMB has also been suggested as a predictive marker of response to immunotherapy in PDAC (Lawlor et al. 2021).

Conflicting results have been observed regarding the activity of checkpoint inhibitors in MSI-H PDAC. In the phase II KEYNOTE-158 trial, the objective response rate with pembrolizumab in the subgroup of 22 PDAC patients was only 18.2%, although the duration of response was not reached at last follow up (Maio et al. 2022). However, various other case reports and series have described impressive and durable responses to immunotherapy in MSI-H PDAC patients (Lemery et al. 2017; Hu et al. 2018; Pothuri et al. 2021; Coston et al. 2023).

To our knowledge, this is the first reported case in literature of a patient with PACC in whom the treatment with pembrolizumab, administered in a later therapy (after almost two years from the treatment start), made possible a surgery approach in an upfront unresectable disease, achieving even a pathological complete response. The pathological complete response rate usually occurs infrequently in pancreatic cancer patients: in a recent real-world report on 274 PDAC patients treated with mFOLFIRINOX or with gemcitabine/nab-paclitaxel combination only 13 (6%) patients achieved a pathological complete response (Macedo et al. 2019). In our PACC patient, these chemotherapeutic regimens failed to control the locally advanced disease, while third-line immunotherapy was able to produce a pathological complete response. Patients with MSI tumors within Lynch syndrome are usually more likely to respond to immune checkpoint inhibitors. The exceptional response in the present case is not explained by a germline mutation in MMR genes instead. Furthermore, our case report suggests that immune checkpoint inhibitor could be more active in dMMR/MSI-H PACC than in dMMR/MSI-H PDAC.

The mutational landscape of PACC is significantly different from PDAC, with a higher proportion of MMR pathway alterations. In the presence of a diagnosis of PACC, it could be advisable to test MSI status and other targetable alterations to orient the therapeutic choice. The activity of immunotherapeutic agents in this setting is suggested to be remarkable.

## Data Availability

Data used during the current study are available from the corresponding author upon reasonable request.

## References

[CR1] Abraham SC, Wu T-T, Hruban RH, Lee J-H, Yeo CJ, Conlon K et al (2002) Genetic and immunohistochemical analysis of pancreatic acinar cell carcinoma: frequent allelic loss on chromosome 11p and alterations in the APC/beta-catenin pathway. Am J Pathol 160:953–962 11891193 10.1016/S0002-9440(10)64917-6 PMC1867188

[CR2] Bergmann F, Aulmann S, Sipos B, Kloor M, von Heydebreck A, Schweipert J et al (2014) Acinar cell carcinomas of the pancreas: a molecular analysis in a series of 57 cases. Virchows Arch 465:661–672 25298229 10.1007/s00428-014-1657-8

[CR3] Butturini G, Pisano M, Scarpa A, D’Onofrio M, Auriemma A, Bassi C (2011) Aggressive approach to acinar cell carcinoma of the pancreas: a single-institution experience and a literature review. Langenbecks Arch Surg 396:363–369 20803029 10.1007/s00423-010-0706-2

[CR4] Calimano-Ramirez LF, Daoud T, Gopireddy DR, Morani AC, Waters R, Gumus K et al (2022) Pancreatic acinar cell carcinoma: A comprehensive review. World J Gastroenterol 28:5827–5844 36353206 10.3748/wjg.v28.i40.5827 PMC9639656

[CR5] Chmielecki J, Hutchinson KE, Frampton GM, Chalmers ZR, Johnson A, Shi C et al (2014) Comprehensive genomic profiling of pancreatic acinar cell carcinomas identifies recurrent RAF fusions and frequent inactivation of DNA repair genes. Cancer Discov 4:1398–1405 25266736 10.1158/2159-8290.CD-14-0617

[CR6] Coston T, Desai A, Babiker H, Sonbol MB, Chakrabarti S, Mahipal A et al (2023) Efficacy of Immune Checkpoint Inhibition and Cytotoxic Chemotherapy in Mismatch Repair-Deficient and Microsatellite Instability-High Pancreatic Cancer: Mayo Clinic Experience. JCO Precis Oncol 7:e2200706 37625102 10.1200/PO.22.00706

[CR7] Cramer S, Marcus MA, Ramkissoon S, Szabo S, Pressey JG (2020) Pediatric BRAF (V600E)-Mutated Pancreatic Acinar Cell Carcinoma With Complete and Durable Response to Dabrafenib and Trametinib. JCO Precis Oncol 4:801–805 35050754 10.1200/PO.19.00343

[CR8] de Wilde RF, Ottenhof NA, Jansen M, Morsink FHM, de Leng WWJ, Offerhaus GJA et al (2011) Analysis of LKB1 mutations and other molecular alterations in pancreatic acinar cell carcinoma. Mod Pathol 24:1229–1236 21572398 10.1038/modpathol.2011.83

[CR9] Distler M, Rückert F, Dittert DD, Stroszczynski C, Dobrowolski F, Kersting S et al (2009) Curative resection of a primarily unresectable acinar cell carcinoma of the pancreas after chemotherapy. World J Surg Oncol 7:22 19239719 10.1186/1477-7819-7-22 PMC2657786

[CR10] FDA (2023) FDA approves pembrolizumab with chemotherapy for HER2-negative gastric or gastroesophageal junction adenocarcinoma. FDA, Paris

[CR11] Florou V, Elliott A, Bailey MH, Stone D, Affolter K, Soares HP et al (2023) Comparative Genomic Analysis of Pancreatic Acinar Cell Carcinoma (PACC) and Pancreatic Ductal Adenocarcinoma (PDAC) Unveils New Actionable Genomic Aberrations in PACC. Clin Cancer Res 29:3408–3417 37266563 10.1158/1078-0432.CCR-22-3724

[CR12] Gaule M, Pesoni C, Quinzii A, Zecchetto C, Casalino S, Merz V et al (2022) Exceptional Clinical Response to Alectinib in Pancreatic Acinar Cell Carcinoma With a Novel ALK-KANK4 Gene Fusion. JCO Precis Oncol 6:e2100400 35005993 10.1200/PO.21.00400 PMC8769132

[CR13] Ghidini M, Lampis A, Mirchev MB, Okuducu AF, Ratti M, Valeri N et al (2021) Immune-Based Therapies and the Role of Microsatellite Instability in Pancreatic Cancer. Genes 2021:12 10.3390/genes12010033 PMC7823781 33383713

[CR14] Gupta M, Sherrow C, Krone ME, Blais EM, Pishvaian MJ, Petricoin EF et al (2021) Targeting the NTRK Fusion Gene in Pancreatic Acinar Cell Carcinoma: A Case Report and Review of the Literature. J Natl Compr Canc Netw 19:10–15 33406492 10.6004/jnccn.2020.7641 PMC8765083

[CR15] Holen KD, Klimstra DS, Hummer A, Gonen M, Conlon K, Brennan M et al (2002) Clinical characteristics and outcomes from an institutional series of acinar cell carcinoma of the pancreas and related tumors. J Clin Oncol 20:4673–4678 12488412 10.1200/JCO.2002.02.005

[CR16] Hu ZI, Shia J, Stadler ZK, Varghese AM, Capanu M, Salo-Mullen E et al (2018) Evaluating Mismatch Repair Deficiency in Pancreatic Adenocarcinoma: Challenges and Recommendations. Clin Cancer Res 24:1326–1336 29367431 10.1158/1078-0432.CCR-17-3099 PMC5856632

[CR17] Ikezawa K, Urabe M, Kai Y, Takada R, Akita H, Nagata S et al (2023) Comprehensive review of pancreatic acinar cell carcinoma: epidemiology, diagnosis, molecular features and treatment. Jpn J Clin Oncol 2023:176 10.1093/jjco/hyad176 PMC10925851 38109477

[CR18] Jiao Y, Yonescu R, Offerhaus GJA, Klimstra DS, Maitra A, Eshleman JR et al (2014) Whole-exome sequencing of pancreatic neoplasms with acinar differentiation. J Pathol 232:428–435 24293293 10.1002/path.4310 PMC4048021

[CR19] Klimstra DS (2007) Nonductal neoplasms of the pancreas. Mod Pathol 20(Suppl 1):S94-112 17486055 10.1038/modpathol.3800686

[CR20] Klimstra DS, Heffess CS, Oertel JE, Rosai J (1992) Acinar cell carcinoma of the pancreas. A clinicopathologic study of 28 cases. Am J Surg Pathol 16:815–837 1384374 10.1097/00000478-199209000-00001

[CR21] Kruger S, Haas M, Burger PJ, Ormanns S, Modest DP, Westphalen CB et al (2016) Acinar cell carcinoma of the pancreas: a rare disease with different diagnostic and therapeutic implications than ductal adenocarcinoma. J Cancer Res Clin Oncol 142:2585–2591 27629876 10.1007/s00432-016-2264-7 PMC11819238

[CR22] Kryklyva V, Haj Mohammad N, Morsink FHM, Ligtenberg MJL, Offerhaus GJA, Nagtegaal ID et al (2019) Pancreatic acinar cell carcinoma is associated with BRCA2 germline mutations: a case report and literature review. Cancer Biol Ther 20:949–955 31002019 10.1080/15384047.2019.1595274 PMC6606020

[CR23] La Rosa S, Adsay V, Albarello L, Asioli S, Casnedi S, Franzi F et al (2012) Clinicopathologic study of 62 acinar cell carcinomas of the pancreas: insights into the morphology and immunophenotype and search for prognostic markers. Am J Surg Pathol 36:1782–1795 23026929 10.1097/PAS.0b013e318263209d

[CR24] La Rosa S, Sessa F, Capella C (2015) Acinar Cell Carcinoma of the Pancreas: Overview of Clinicopathologic Features and Insights into the Molecular Pathology. Front Med (lausanne) 2:41 26137463 10.3389/fmed.2015.00041 PMC4469112

[CR25] Lack EE, Cassady JR, Levey R, Vawter GF (1983) Tumors of the exocrine pancreas in children and adolescents. A clinical and pathologic study of eight cases. Am J Surg Pathol 7:319–327 6307069 10.1097/00000478-198306000-00003

[CR26] Lawlor RT, Mattiolo P, Mafficini A, Hong S-M, Piredda ML, Taormina SV et al (2021) Tumor Mutational Burden as a Potential Biomarker for Immunotherapy in Pancreatic Cancer: Systematic Review and Still-Open Questions. Cancers (basel). 2021:13 10.3390/cancers13133119 PMC8269341 34206554

[CR27] Lemery S, Keegan P, Pazdur R (2017) First FDA Approval Agnostic of Cancer Site - When a Biomarker Defines the Indication. N Engl J Med 377:1409–1412 29020592 10.1056/NEJMp1709968

[CR28] Li M, Mou Y, Hou S, Cao D, Li A (2018) Response of germline BRCA2-mutated advanced pancreatic acinar cell carcinoma to olaparib: A case report. Medicine (baltimore) 97:e13113 30407325 10.1097/MD.0000000000013113 PMC6250555

[CR29] Liu W, Shia J, Gönen M, Lowery MA, O’Reilly EM, Klimstra DS (2014) DNA mismatch repair abnormalities in acinar cell carcinoma of the pancreas: frequency and clinical significance. Pancreas 43:1264–1270 25058881 10.1097/MPA.0000000000000190

[CR30] Lowery MA, Goff LW, Keenan BP, Jordan E, Wang R, Bocobo AG et al (2019) Second-line chemotherapy in advanced biliary cancers: A retrospective, multicenter analysis of outcomes. Cancer 125:4426–4434 31454426 10.1002/cncr.32463 PMC8172082

[CR31] Luchini C, Scarpa A (2023) Microsatellite instability in pancreatic and ampullary carcinomas: histology, molecular pathology, and clinical implications. Hum Pathol 132:176–182 35714836 10.1016/j.humpath.2022.06.009

[CR32] Macedo FI, Ryon E, Maithel SK, Lee RM, Kooby DA, Fields RC et al (2019) Survival Outcomes Associated With Clinical and Pathological Response Following Neoadjuvant FOLFIRINOX or Gemcitabine/Nab-Paclitaxel Chemotherapy in Resected Pancreatic Cancer. Ann Surg 270:400–413 31283563 10.1097/SLA.0000000000003468 PMC9634701

[CR33] Maio M, Ascierto PA, Manzyuk L, Motola-Kuba D, Penel N, Cassier PA et al (2022) Pembrolizumab in microsatellite instability high or mismatch repair deficient cancers: updated analysis from the phase II KEYNOTE-158 study. Ann Oncol 33:929–938 35680043 10.1016/j.annonc.2022.05.519

[CR34] Mandelker D, Marra A, Zheng-Lin B, Selenica P, Blanco-Heredia J, Zhu Y et al (2023) Genomic Profiling Reveals Germline Predisposition and Homologous Recombination Deficiency in Pancreatic Acinar Cell Carcinoma. J Clin Oncol 41:5151–5162 37607324 10.1200/JCO.23.00561 PMC10667000

[CR35] Moore PS, Orlandini S, Zamboni G, Capelli P, Rigaud G, Falconi M et al (2001) Pancreatic tumours: molecular pathways implicated in ductal cancer are involved in ampullary but not in exocrine nonductal or endocrine tumorigenesis. Br J Cancer 84:253–262 11161385 10.1054/bjoc.2000.1567 PMC2363700

[CR36] Mosele F, Remon J, Mateo J, Westphalen CB, Barlesi F, Lolkema MP et al (2020) Recommendations for the use of next-generation sequencing (NGS) for patients with metastatic cancers: a report from the ESMO Precision Medicine Working Group. Ann Oncol 31:1491–1505 32853681 10.1016/j.annonc.2020.07.014

[CR37] Pothuri V, Herndon J, Ballentine SJ, Lim K-H, Fields RC (2021) A Case of a Pathological Complete Response to Neoadjuvant Nivolumab plus Ipilimumab in Periampullary Adenocarcinoma. Oncologist 26:722–726 33982365 10.1002/onco.13821 PMC8417855

[CR38] Qu Q, Xin Y, Xu Y, Yuan Y, Deng K (2022) Imaging and Clinicopathological Features of Acinar Cell Carcinoma. Front Oncol 12:888679 35747811 10.3389/fonc.2022.888679 PMC9209696

[CR39] Sakakida T, Ishikawa T, Doi T, Morita R, Kataoka S, Miyake H et al (2023) Genomic landscape and clinical features of rare subtypes of pancreatic cancer: analysis with the national database of Japan. J Gastroenterol 58:575–585 37029223 10.1007/s00535-023-01986-9 PMC10199859

[CR40] Schmid M (1957) The syndrome of metastasizing, exocrine pancreas adenoma with secretory activity. Z Klin Med 154:439–455 13456796

[CR41] Schmidt CM, Matos JM, Bentrem DJ, Talamonti MS, Lillemoe KD, Bilimoria KY (2008) Acinar cell carcinoma of the pancreas in the United States: prognostic factors and comparison to ductal adenocarcinoma. J Gastrointest Surg 12:2078–2086 18836784 10.1007/s11605-008-0705-6

[CR42] Takahashi H, Ikeda M, Shiba S, Imaoka H, Todaka A, Shioji K et al (2021) Multicenter Retrospective Analysis of Chemotherapy for Advanced Pancreatic Acinar Cell Carcinoma: Potential Efficacy of Platinum- and Irinotecan-Containing Regimens. Pancreas 50:77–82 33370026 10.1097/MPA.0000000000001718 PMC7748047

[CR43] Yoo C, Kim BJ, Kim K-P, Lee J-L, Kim TW, Ryoo B-Y et al (2017) Efficacy of Chemotherapy in Patients with Unresectable or Metastatic Pancreatic Acinar Cell Carcinoma: Potentially Improved Efficacy with Oxaliplatin-Containing Regimen. Cancer Res Treat 49:759–765 27857025 10.4143/crt.2016.371 PMC5512358

[CR44] https://www.ema.europa.eu/en/documents/product-information/keytruda-epar-product-information_en.pdf.

